# Association between *ZASP/LDB3* Pro26Ser and Inclusion Body Myopathy

**DOI:** 10.3390/ijms25126547

**Published:** 2024-06-14

**Authors:** Daniela Piga, Simona Zanotti, Michela Ripolone, Laura Napoli, Patrizia Ciscato, Sara Gibertini, Lorenzo Maggi, Francesco Fortunato, Andrea Rigamonti, Dario Ronchi, Giacomo Pietro Comi, Stefania Corti, Monica Sciacco

**Affiliations:** 1 Neurology Unit, Fondazione IRCCS Ca’ Granda Ospedale Maggiore Policlinico, 20122 Milan, Italy; 2 Neuromuscular and Rare Disease Unit, Fondazione IRCCS Ca’ Granda Ospedale Maggiore Policlinico, 20122 Milan, Italy; 3 Neuroimmunology and Neuromuscular Diseases Unit, Fondazione IRCCS Istituto Neurologico “Carlo Besta”, 20133 Milan, Italy; 4 Dino Ferrari Center, Department of Pathophysiology and Transplantation, University of Milan, 20122 Milan, Italy; 5 UOC Neurologia–Stroke Unit, Presidio “A. Manzoni”, ASST Lecco, 23900 Lecco, Italy

**Keywords:** ZASP/LDB3, inclusion body myositis, rimmed vacuoles, core-like alterations, next-generation sequencing

## Abstract

Inclusion body myositis (IBM) is a slowly progressive disorder belonging to the idiopathic inflammatory myopathies, and it represents the most common adult-onset acquired myopathy. The main clinical features include proximal or distal muscular asymmetric weakness, with major involvement of long finger flexors and knee extensors. The main histological findings are the presence of fiber infiltrations, rimmed vacuoles, and amyloid inclusions. The etiopathogenesis is a challenge because both environmental and genetic factors are implicated in muscle degeneration and a distinction has been made previously between sporadic and hereditary forms. Here, we describe an Italian patient affected with a hereditary form of IBM with onset in his mid-forties. Next-generation sequencing analysis disclosed a heterozygous mutation c.76C>T (p.Pro26Ser) in the PDZ motif of the *LDB3/ZASP* gene, a mutation already described in a family with a late-onset myopathy and highly heterogenous degree of skeletal muscle weakness. In the proband’s muscle biopsy, the expression of ZASP, myotilin, and desmin were increased. In our family, in addition to the earlier age of onset, the clinical picture is even more peculiar given the evidence, in one of the affected family members, of complete ophthalmoplegia in the vertical gaze. These findings help extend our knowledge of the clinical and genetic background associated with inclusion body myopathic disorders.

## 1. Introduction

Sporadic inclusion body myositis (sIBM) represents the most common form of idiopathic inflammatory myopathy among individuals, predominantly males, above 50 years of age [1,2]. Its distinctive clinical features include slowly progressive skeletal muscle weakness and atrophy with a major involvement of quadriceps and long finger flexors [3]. IBM is a degenerative disorder that ultimately leads to loss of ambulation. Also, nutritional and respiratory complications may occur when oropharyngeal muscles are involved [4]. Creatine kinase levels are slightly elevated, and electromyography reveals myogenic abnormalities or mixed neurogenic and myogenic changes [3]. Skeletal muscle morphological observation shows degenerative features—namely, rimmed vacuoles, accumulation of myotoxic proteins/products including amyloid, TDP43 (TAR DNA binding protein 43), and p62, and, at the ultrastructural level, myonuclear degeneration and cytoplasmic or nuclear tubulofilaments—as well as inflammatory signs like fiber cellular infiltrations and upregulation of MHC (major histocompatibility complex). This pathologic process can lead to mitochondrial dysfunction resulting in cytochrome c oxidase deficiency and evidence of ragged red fibers.

In addition, to make IBM etiopathogenesis even more challenging, several both autosomal-recessive and -dominant cases have been reported. These groups of myopathies share their histological features with sIBM except for the lack of MHC upregulation. For this reason, they are more properly classified as Hereditary Inclusion Body Myopathies (hIBM) [5]. Clinically, hIBM cases are highly heterogeneous and manifest at an earlier age of onset. A number of different genes have been so far identified and associated with distinctive hIBM clinical manifestations including both the peripheral and/or the central nervous system [2,6].

We describe an Italian patient, now aged 56 years, reporting an 11-year history of slowly progressive, mainly proximal and axial myopathy without any cardiac involvement, whose histopathological features meet the criteria of hIBM. Next generation sequence (NGS) analysis disclosed a heterozygous missense pathogenic variant in the PDZ motif of the *LDB3/ZASP* gene.

Indeed, a very recent revision (2024) of the diagnostic IBM criteria has changed the old terminology eliminating the terms “sporadic”, “familial”, and “hereditary [7]. Following these criteria, we more properly refer to our case as an inclusion body ZASP-associated myopathy.

ZASP (Z-band alternatively spliced PDZ-motif protein) is a sarcomeric protein encoded by the *LDB3* gene (NM_007078.2) and expressed in skeletal and cardiac muscle at Z-disc. ZASP, also known as LDB3 (LIM domain binding 3), belongs to an enigmatic family as it plays an important role in the stabilization of the sarcomeric structure [8] and is involved in the signaling pathway of striated muscle [9]. In addition to an N-term conserved PDZ domain, mammalian ZASP has either one ZM (Zasp-like motif) domain or three LIM (Lin-11 Isl-1 Mec-3) domains [10]. All these structural domains mediate the protein complexes assembling in the muscle Z-disc by direct interactions: in particular, the PDZ region binds to the C-term of α-actinin, myotilin, and myozenin [11], while the ZM region is involved in the binding with actin and the self-interaction with other ZASP proteins [12].

Point mutations in the human *LDB3/ZASP* have been associated with various cardiac and skeletal muscle disorders such as dilated cardiomyopathy (DCM) [13], left ventricular non-compaction (LVNC) [13], hypertrophic cardiomyopathy [14], and sporadic inclusion body myositis [15]. Mutations in *LDB3* were also found in patients with an autosomal dominant form of progressive muscular dystrophy, with histopathological features of myofibrillar myopathies (MFMs) termed zaspopathy [16,17]. MFMs refer to a group of muscle disorders characterized by myofibrillar dysfunction caused by mutations in a variety of Z-line proteins. Mutations in *ZASP* responsible for MFMs are localized primarily at ZM-motif and LIM domains [18].

The identification of another family carrying the missense mutation Pro26Ser in the PDZ domain of the ZASP protein allows the clinical spectrum associated with both the mutation itself and the already-known phenotypic IBM heterogeneity to be extended.

## 2. Results and Discussion

A 56-year-old male sought medical observation at the age of 48 years following a three-year history of difficulty in climbing stairs and in rising from a chair or from the floor. He did not refer to any upper limb difficulties, nor did he experience dysphagia. He performed a sedentary job and had never practiced any sports activity. Serum creatine kinase (CK) levels were only slightly elevated, never higher than 259 U/L (normal values = 26–192).

Needle electromyography (EMG) showed myopathic signs in the lower limb muscles and a normal electroneuronographic examination. Over the years, proximal myopathic signs were detected in both upper and lower limbs as well as axially and in facial muscles.

Skeletal muscle Magnetic Resonance Imaging (MRI) at the age of 48 years showed adipose degeneration and hypotrophy affecting the quadriceps and thigh muscles, with relative sparing of the adductors and of the rectus femoris, and minimal involvement of the leg muscles with sparing of the tibialis anterior and peroneals. Edema was detected at the vastus lateralis of the quadriceps muscles. Five years later, moderate to severe fibroadipose degeneration was detected at the pelvic girdle, especially gluteus and tensor fascia lata, and in all thigh muscles, still with relative sparing of the adductors and of the rectus femoris and with resolution of the edema. Muscle infiltration also involved calf muscles, with mild hypotrophy of medial gastrocnemius, and all shoulder girdle muscles.

Clinically, the progression of the disease was slow, even though the patient reported a few falls, especially when walking on uneven terrain. When he falls, he can get back to a standing position independently only if he has support. He can climb stairs only one step at a time and with the help of handrails. He denies upper limb problems, diplopia/ptosis, dysphagia, or dyspnea. Cardiac examination and respiratory tests remain normal. At a recent (March 2024) neurological examination, he could walk normally with minimal difficulties on toes, he had no winged scapulae, tendon retractions, or pyramidal signs. He had no facial involvement except for the presence of minimal limitation in the upward conjugated eye movements. He could rise from a chair with the help of both hands but was unable to perform squats. Medical Research Council (MRC) evaluation of single muscle/muscle groups disclosed weakness at neck flexion (3.5/5), elbow flection (4+/5) and extension (3+/5), iliopsoas (2+/5), knee extension (4−/5) and flexion (4/5), and foot dorsiflexion (4+/5). Mild hypotrophy of the distal quadriceps was also evident.

**Figure 1:**
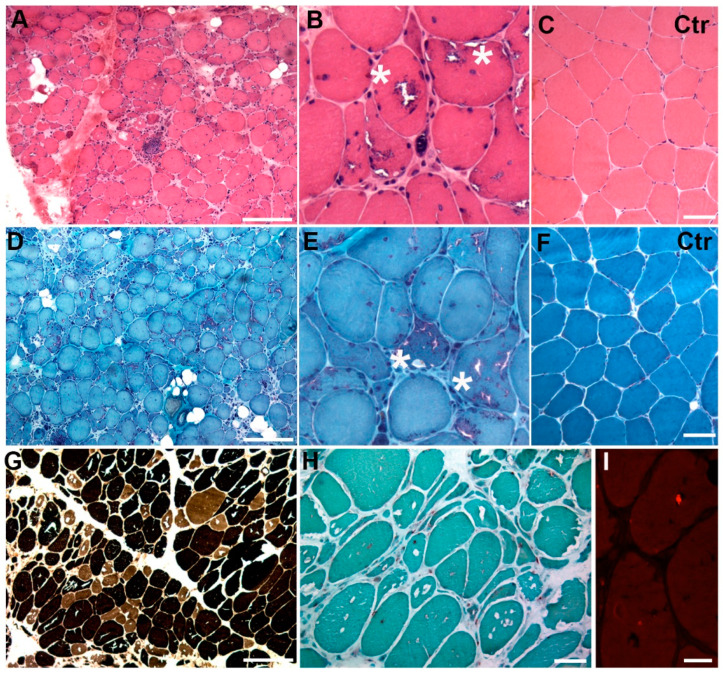
H&E (**A**,**B**) and MTG (**D**,**E**) in the proband shows increased fiber size variability and numerous fibers with rimmed vacuoles (asterisks). ATPase staining at pH 4.6 (**G**) shows a prevalence of type I fibers, whereas hypotrophic fibers belong to both fiber types. Vacuoles were detected in both type I and II fibers. FA activity (**H**) is increased in the cytoplasm of some fibers. Fluorescence Congo red (**I**) staining shows an amyloid deposit within a vacuole. (**C**,**F**): H&E and MTG staining in a control sample. Scale bar 50 µm.

Right quadriceps (lateral vastus) skeletal muscle biopsy showed a marked fiber size variability with several centralized nuclei and rare fiber splitting. The most prominent histopathological feature was the presence of many fibers with rimmed vacuoles of variable size localized in either the cytoplasm or at subsarcolemmal level in both type I and type II fibers. A marked increase in endo- and perimysial connective tissue was also observed along with a slight increase in interstitial cellularity. Acid phosphatase activity was moderately increased in the cytoplasm of some hypotrophic fibers and, rarely, in rimmed vacuoles. Congo Red immunofluorescence allowed the detection of amyloid deposits in a few vacuoles. Glycogen and lipid content were normal as well as cytochrome oxidase (COX), succinate dehydrogenase (SDH), and nicotinamide adenine dinucleotide diaphorase (NADH) activities (Figure 1 and Figure S1). The reactions for both MHC-I and C5b9 were negative, indicating a lack of inflammation.

**Figure 2:**
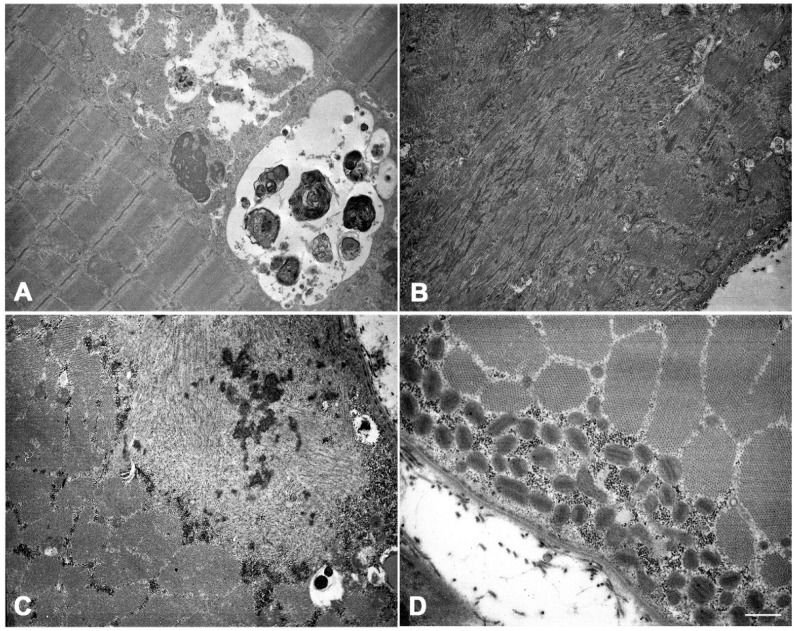
Ultrastructural examination shows autophagic vacuoles containing degraded membranous material and myelinoid bodies (**A**), core-like areas displaying myofibrillar disarray and Z-line streaming (**B**), disorganized regions with electron-dense granulofilamentous masses (**C**), and an accumulation of mitochondria, some of which with paracrystalline inclusions (**D**). Scale bar: (**A**,**B**): 2.27 µm. (**C**,**D**): 0.83 µm.

Ultrastructural examination revealed autophagic vacuoles containing osmiophilic membrane debris and myelinoid bodies, loose glycogen deposits, and subsarcolemmal accumulation of mitochondria, some of which with paracrystalline inclusions (Figure 2A,D). Additionally, in a few muscle fibers, we observed large, focal, core-like alterations in the sarcomeric architecture, displaying myofibrillar disarray and Z-band streaming (Figure 2B). In other fibers, we identified areas with completely disorganized filaments, within which pathological aggregates with an electrondense granulofilamentous appearance accumulated (Figure 2C).

**Figure 3:**
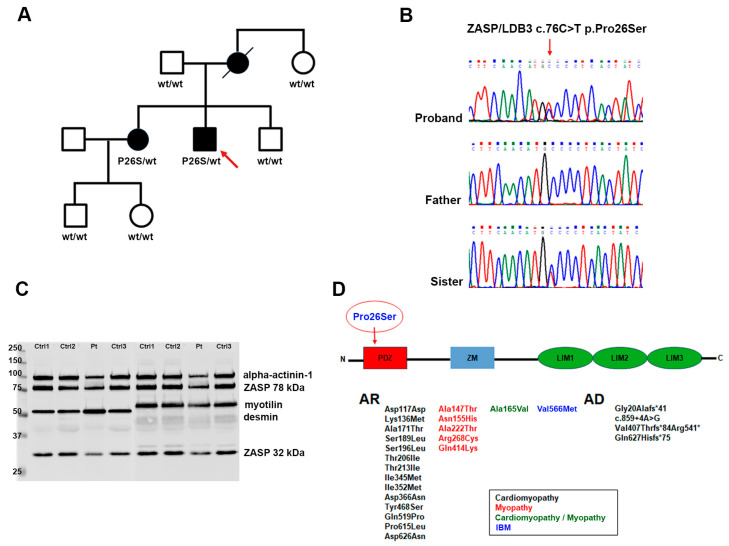
Family pedigree with black symbols indicating the affected members. The arrow indicates the proband (**A**). Electropherograms in the proband, his sister, and their father (**B**). Representative Western blot of controls and patient samples. Lane 1–4 staining with LDB3 (78 KDa and 32 KDa) and desmin. Lane 5–8 staining with LDB3 and myotilin. α-actinin-1 was used as loading control (**C**). Schematic representation of LDB3 protein structure with the indication of the already reported variants. The arrow shows the localization of our patient’s mutation in the PDZ domain (**D**).

These skeletal muscle features were consistent with a diagnosis of hIBM, for which reason we performed NGS genetic analysis using a target gene panel for vacuolar, distal, and myofibrillar myopathies. This study led to the identification of a heterozygous dominant missense mutation in LDB3/ZASP, namely the c.76C>T replacement that causes the p.Pro26Ser protein alteration of a highly conserved residue. The variant is located in the PDZ domain, which is predicted to be involved in the Z-line integrity of skeletal muscle because of its interactions with α-actinin-2 and myotilin (Figure 3) [9].

It has been reported that because ZASP acts like an adaptor protein recruiting multiple factors at the Z-disc and maintaining regular and precise arrangements of sarcomeric actin filaments, the mutation could sequestrate F-actin cross-linking proteins [10]. In order to establish if there was an alteration in ZASP and other myofibrillar proteins, we performed a Western blot analysis evaluating the expression of ZASP, desmin, and myotilin. The production of ZASP, revealed by anti-LDB3 antibody and normalized on α-actinin-1, displayed a slight increase in the high 78 kDa isoform (1.93 ± 0.35 arbitrary units (AU) in patients compared to 1.59 ± 0.189 AU in controls; *p* = 0.2636), whereas the low 32 kDa band was more similar to that observed in the control samples (0.72 ± 0.17 AU in patients compared to 0.56 ± 0.06 AU in controls; *p* = 0.428). Conversely, both desmin and myotilin were significantly increased (Figure 3C). In detail, densitometric analysis for desmin showed a value of 358.4 ± 24.55 AU in patients and 89.62 ± 4.26 AU in controls; *p* = 0.0091); for myotilin, a value of 145.7 ± 9.11 AU for patients and 89.49 ± 3.93 AU for controls; *p* = 0.0091). This observation was also confirmed in previous studies indicating that patients with zaspopathies had significant myotilin accumulations in their skeletal muscle fibers [17].

The same molecular defect has been recently reported as pathogenic in a familiar case of late-onset (mid-fifties) myopathy, with distal, proximal, and axial muscle involvement [19]. Clinical, radiological, histological, and ultrastructural features of the reported case are very similar to those of our patient. Also, similarly, the expression of the 78 kDa ZASP isoform and desmin was increased. In that family, the mutation was identified also in the proband’s, subjectively asymptomatic, elder sister, whose neurological examination revealed moderate signs of disto-proximal myopathy (difficulty in walking on heels and toes, weakness of the extensor digitorum communis and psoas muscles) as well as mild weakness in neck flexors, a condition confirmed by muscle Computed Tomography (CT) scan, which showed evidence of muscle atrophy with associated fatty replacement.

We clinically and genetically evaluated our proband’s family members, all of them reportedly asymptomatic, namely, the 88 y.o. father, the 80 y.o. maternal aunt, the 49 y.o. younger brother, the 60 y.o. elder sister, and the son and daughter of the latter, aged 37 and 34 years, respectively (Figure 3A). The proband has no siblings, parental consanguinity was excluded. The mother died in 2019 at the age of 83 years following acute septicemia, she had been in a wheelchair for over 30 years due to left hemiplegia caused by a rupture of a brain aneurysm. Her relatives state that she was getting worse in the last years of life; however, they are unable to specify whether she had any additional movement issues. The mutation was only detected in the patient’s sister, who did not reference any skeletal muscle problems, indeed, she practices skiing, trekking, and biking activities on a weekly basis, and she denies any symptoms during or after these activities except for the presence of diffuse, mild myalgia, never associated with myoglobinuria, after intense and/or long-lasting efforts (Figure 3B). Her serum CK levels are normal. Neurological examination, however, revealed discrete, selected bilateral weakness at elbow extension (MRC 4/5) and flexion (MRC 4.5/5). Also, she has complete bilateral vertical gaze ophthalmoplegia, without ptosis, which was not detected in the other non-mutated family members. 

Our findings confirm the deleterious effect of the c.76C>T variant, which is liable to be classified as likely pathogenic in the ACGM guidelines.

Despite the clinical and histopathological similarities between the two families harboring the same mutation, including an intriguing family tree in which the proband is a severely affected male with an elder, mildly affected sister, some substantial differences need to be underlined. To begin with, in our case, the disease onset (mid-forties) is at least ten years earlier compared to the other reported cases. Also, our family shows a peculiar and selective involvement of conjugated eye movements, which is particularly severe—complete vertical gaze palsy—in the proband’s sister, who is, on the other hand, only mildly affected in terms of limb and axial involvement. Given the peculiarity of the neurological abnormality, we were unable to establish if the ophthalmoparesis is congenital or exactly when it started in both the proband and her sister, nor are we able to predict, at the moment, whether the defect is progressive. Clinical follow-up will possibly help clarify this issue. Supplementary Table S1 shows a clinical comparison between our family and the previously reported one sharing the same *LDB3* variant.

Only one other mutation in *ZASP/LDB3*, the missense c.1719G>A (p.V566M), has been so far associated with a case of apparently sporadic IBM [15]. This alteration was localized in the LIM3 domain of LDB3, which links the protein kinase C, and therefore it is potentially involved in the signaling pathway of striated muscle [20]. The affected subject was a 63 y.o. woman with a 23-year history of slowly progressive, mainly distal myopathy, normal serum CK levels, and no history of heart disease. The IBM diagnosis was made on the basis of skeletal muscle biopsy findings showing slight to moderate evidence of rimmed vacuoles along with mild inflammatory infiltration.

Until recently, the majority of ZASP/LDB3 mutations have been linked to cardiomyopathies and ZASP-related myofibrillar myopathies (Figure 3D). For both these disorders, mutations seem to be concentrated in the ZM domain and in the LIMs regions [18]. The missense p.Pro26Ser variant is the first identified in the PDZ domain and, even if the observation is limited to only two families and to a restricted number of affected subjects, it seems to be associated with a highly variable age of onset and clinical heterogeneity with no evidence of cardiac involvement.

We believe this investigation has contributed to expanding our knowledge on the phenotypes associated with IBMs, more specifically so to those caused by ZASP mutations and thus simultaneously characterized by an alteration of myofibrillar proteins. Further studies, including genetic background analyses and/or detection of modifying genes, are certainly required to clarify the reasons for the observed clinical heterogeneity and severity. However, this IBM/MFM genetic association expands the scenario of the patho-mechanisms underlying neuromuscular diseases, which remains very complex and challenging. Studies of larger cohorts as well as the discovery of other pathogenic variants by WES and WGS will certainly shed light on this group of rare diseases.

## 3. Materials and Methods

### 3.1. Muscle Biopsy

#### 3.1.1. Light Microscopy

Skeletal muscle biopsy from right vastus lateralis was performed at Neuromuscular and Rare Disease Unit. Tissue specimen was frozen in isopentane-cooled liquid nitrogen and processed according to standard techniques, as previously described [21]. For histological analysis, 8 µm thick cryo-sections were picked and processed for routine staining with Hematoxylin and Eosin (H&E), Modified Gomori Trichrome (MGT), myosin ATPase (pH 9.4-4.6-4.3), cytochrome c oxidase (COX), succinate dehydrogenase (SDH), phosphatase acid (PA), NADH, Oil Red O, Periodic Acid Schiff (PAS), and Congo Red [22].

#### 3.1.2. Electron Microscopy

For ultrastructural examination, a small part of muscle sample was fixed in 2.5% glutaraldehyde (Electron Microscopy Sciences EMS, Hatfield, PA, USA) for 1 h at room temperature and O.N. at 4 °C, the specimens were washed in 0.1 M, pH 7.4 cacodylate buffer (EMS), post-fixed for 1 h in 2% osmium tetroxide (EMS). They were then rinsed again with the cacodylate buffer (4 for 5 min). Next, the specimens were dehydrated using a graded series of ethyl alcohol and twice with oxide propylene (Sigma-Aldrich, Burlington, MA, USA); after dehydration, the samples were embedded in Epon’s resin (EMS). Finally, ultrathin sections (80 nm thick slices) were prepared using an ultramicrotome Power Tome XL (RMC, Tucson, AZ, USA). The grids, stained with 0.5% lead citrate (EMS) and uranyl acetate replacement/methanol 1:1 (EMS), were examined with Zeiss EM109 transmission electron microscope (Carl Zeiss, Oberkochen, Germany).

#### 3.1.3. Western Blot

Western blotting was performed on muscle homogenates from patients and three age-matched controls. The latter are subjects who had undergone muscle biopsy for suspected neuromuscular disease, but whose muscle biopsy had turned out normal.

Briefly, 30 mg of samples was electrophoresed on 10% SDS-PAGE and transferred onto nitrocellulose membranes. Membranes were probed with antibodies to LDB3 (1:15,000, goat polyclonal; ab110003 Abcam, Cambridge, UK), to myotilin (1:250, mouse monoclonal; Novocastra, Newcastle upon Tyne, UK), to desmin (1:200, mouse monoclonal; Novocastra, Newcastle upon Tyne, UK), and α-actinin-1 (1:5000; Sigma-Aldrich, Burlington, MA, USA). α-actinin-1 was used as an indicator of protein loading. Blocked membranes were then incubated in IRDye secondary antibodies (1:18,000; Li-Cor, Lincoln, NE, USA) and bands were detected and quantified with the Odyssey Fc Imaging System (Li-Cor, Lincoln, NE, USA). Immunobands were quantitated densitometrically as arbitrary unit (AU) using n-Image Studio Software (Li-COR Biosciences, Bad Homburg, Germany). Statistical analysis was performed using GraphPad Prism 10.2.3 (GraphPad Software Inc., La Jolla, CA, USA). Data are expressed mean ± SEM.

### 3.2. Genetic Testing

A written informed consent was obtained from the proband and the examined family members. Genomic DNA was extracted from the peripheral blood using Freedom Evo 100 (Tecan, Mannedorf, Switzerland) by Nucleo Spin blood Kit following the manufacturer’s instructions (Macherey–Nagel, Düren, Germany). DNA quality and quantity were analyzed by NanoDrop (Thermo Fisher, Waltham, MA, USA), gel electrophoresis, and fluorescence absorbance (Qubit^®^ 2.0 Fluorometer; Thermo Fisher). We performed a custom target gene panel testing for vacuolar, distal, and myofibrillar myopathies by Next Generation Sequencing (NGS) approach, designed with Agilent’s HaloPlex technology (Agilent Technologies, Santa Clara, CA, USA) loaded on Illumina MiSeq sequencer. In particular, regions of interest (ROI) were defined as coding sequences and flanking splice consensus sequences from genes (padding: +/− 50 bp). The required coverage for any sequenced region is set to 300 reads. Copy Number Variation (CNV) and Coverage data were obtained from BAM files and bed files with bedtools2 on Linux platform. To annotate functional consequences of genetic variation, we used the free software wANNOVAR (https://wannovar.wglab.org (accessed on 2 February 2024)). The annotated variants are filtered based on frequencies in the available databases (1000 Genome Project, GnomAD browser, and Exome Variant Server) and prioritized by clinical indication. Pathogenicity of unknown variants was predicted using online tools like Panther, Polyphen2, MutPred, Sift, and Mutation Taster. All the mutations with clinical relevance were confirmed by Sanger sequencing using Big Dye Terminator on the ABI 3100XL (Life Technologies, Carlsbad, CA, USA). This study led to the identification of the c.76C>T heterozygous missense mutation in LDB3/ZASP. The segregation analysis was performed on all the available family members (Figure 3A). The substitution was searched in the Franklin database (https://franklin.genoox.com/ (accessed on 12 February 2024)) and was classified as VUS. However, according to the ACMG guidelines [23], it met the PM2 criteria for moderate pathogenic variants, because the frequency of this missense in gnomAD population databases was extremely low with a MAF (minimum allele frequency) value of less than 1%.

## Data Availability

All data generated or analyzed during this study are included in this published article.

## Supplementary Materials

The following supporting information can be downloaded at: https://www.mdpi.com/article/10.3390/ijms25126547/s1.
