# Supplementary figures and images for: Association between ZASP/LDB3 Pro26Ser and Inclusion Body Myopathy
Source: Int J Mol Sci. 2024 Jun 14;25(12):6547. doi: 10.3390/ijms25126547 (PMC11203685; doi:10.3390/ijms25126547)

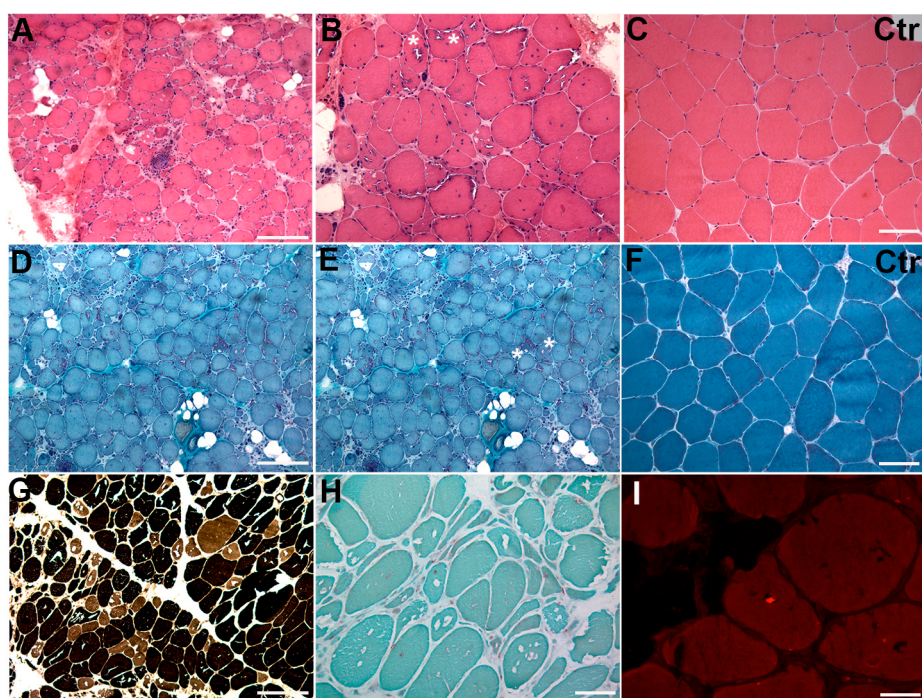

**Figure S1.** Original uncut size of images B, C, E, F and I of Figure 1.

Supplement: Supplementary file 1 [file ijms-25-06547-s001.zip › Figure S1.pdf]
